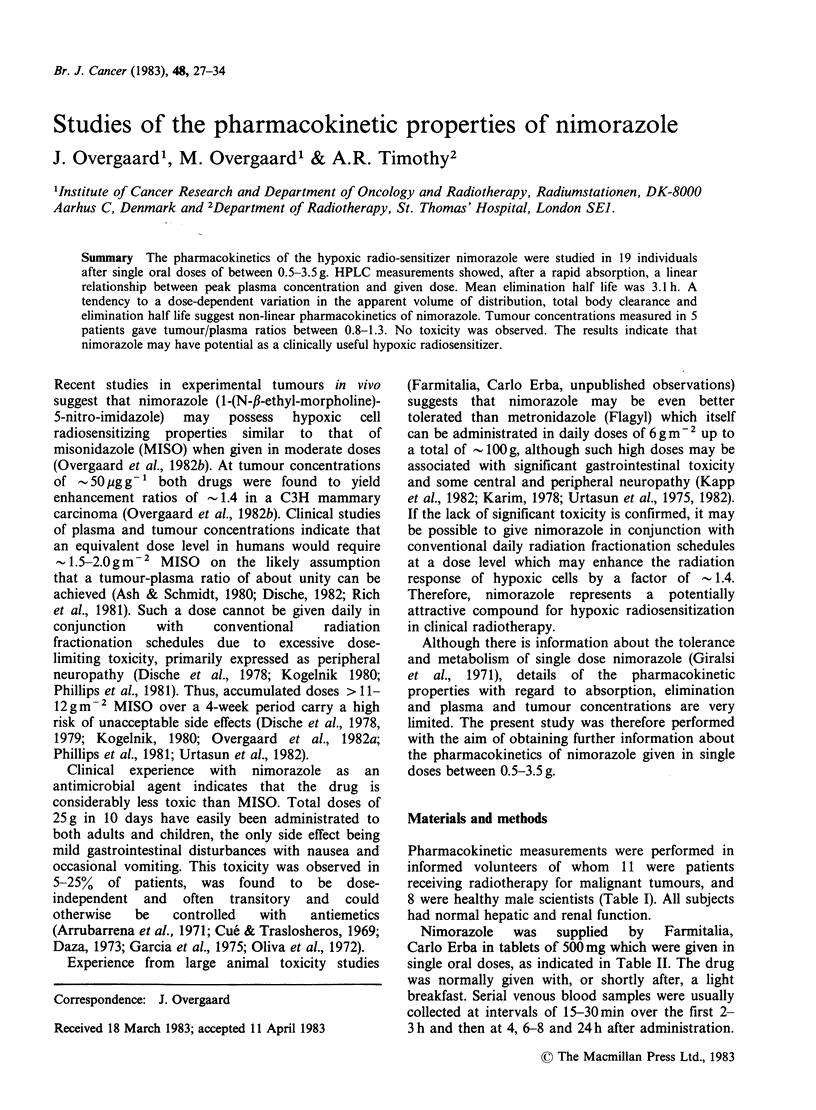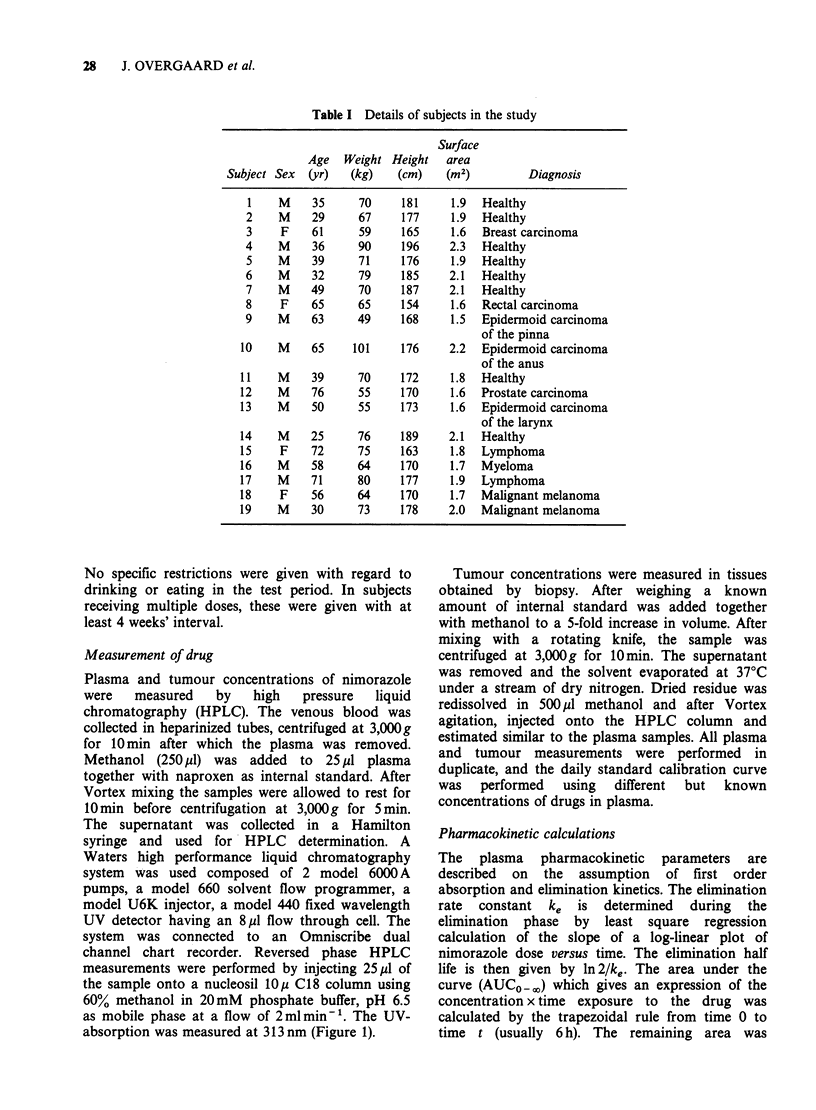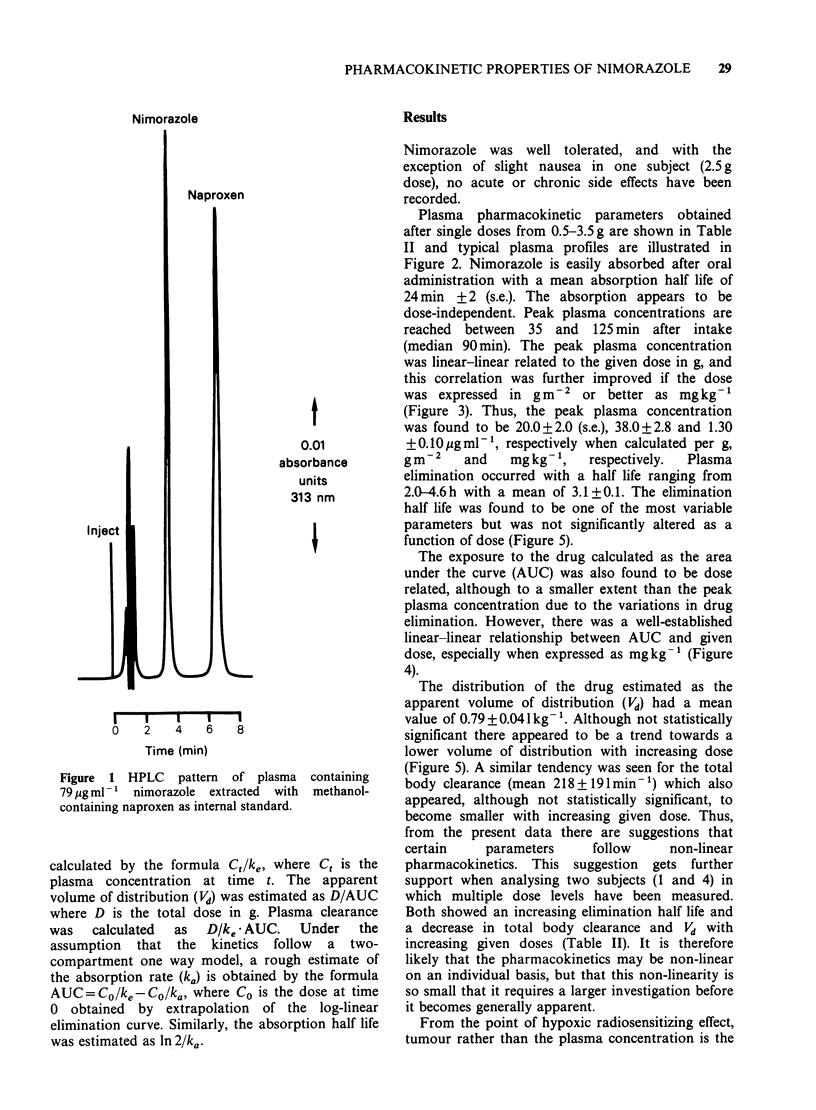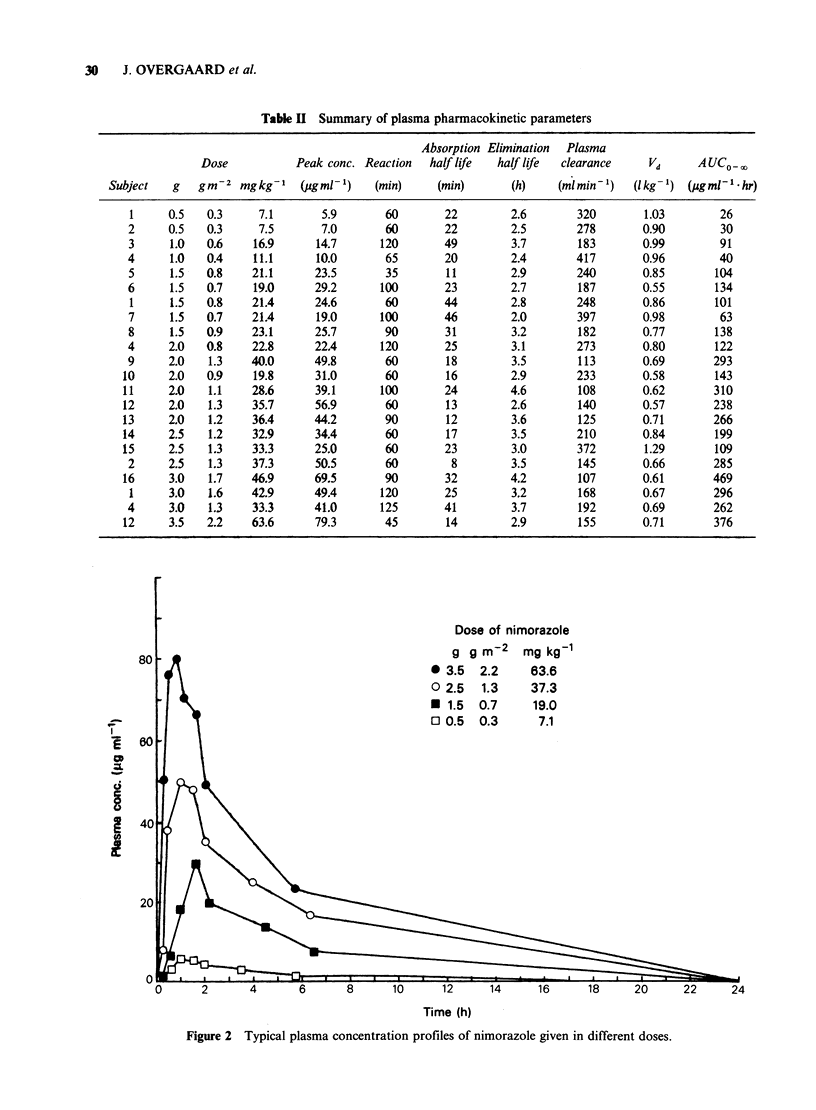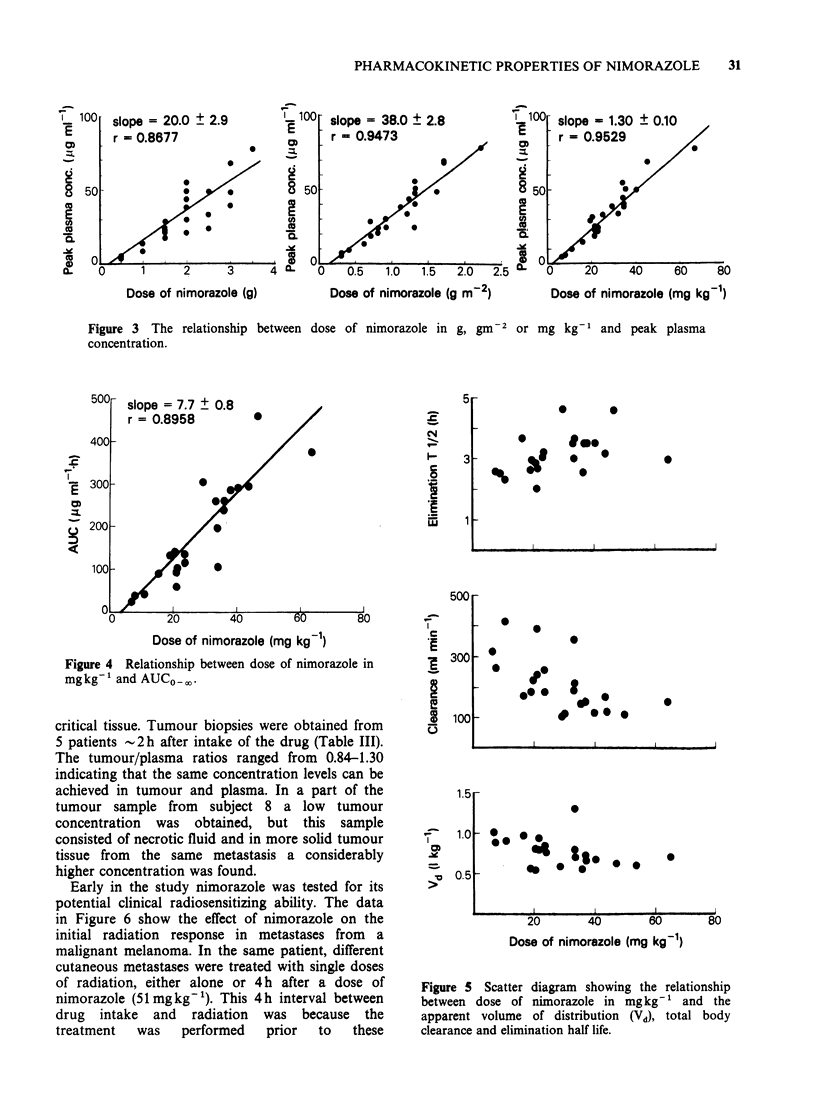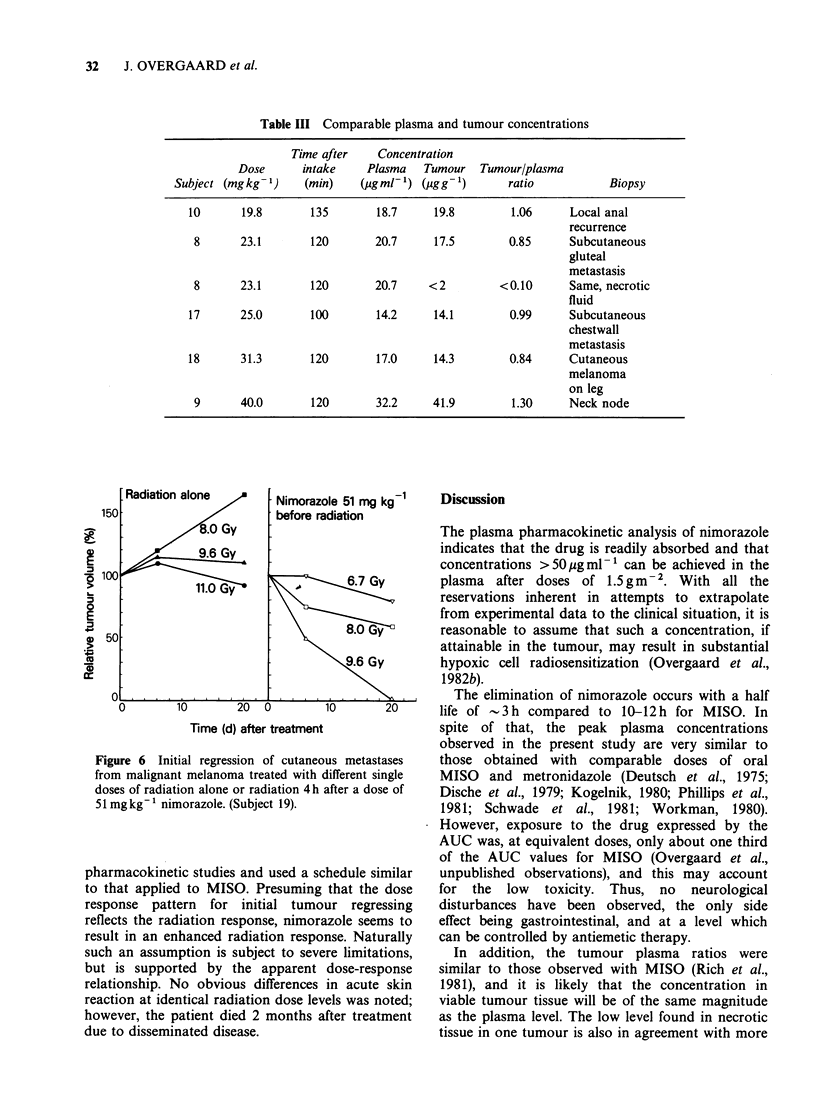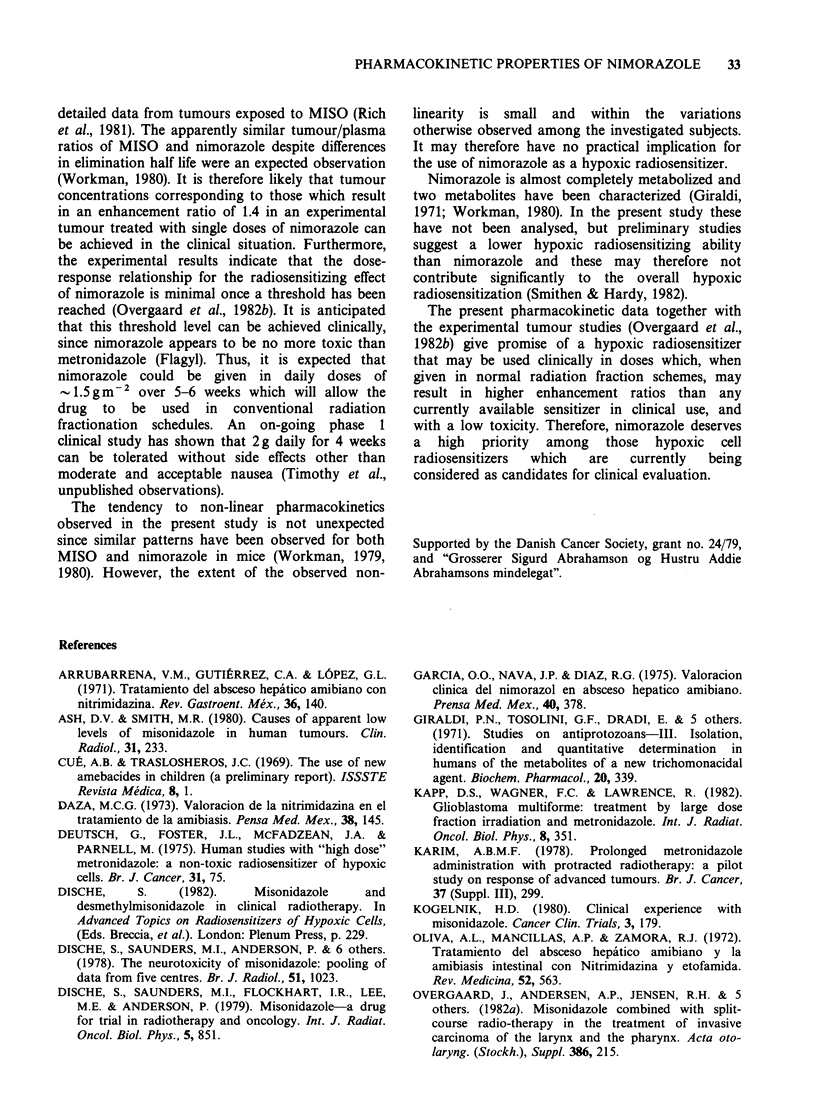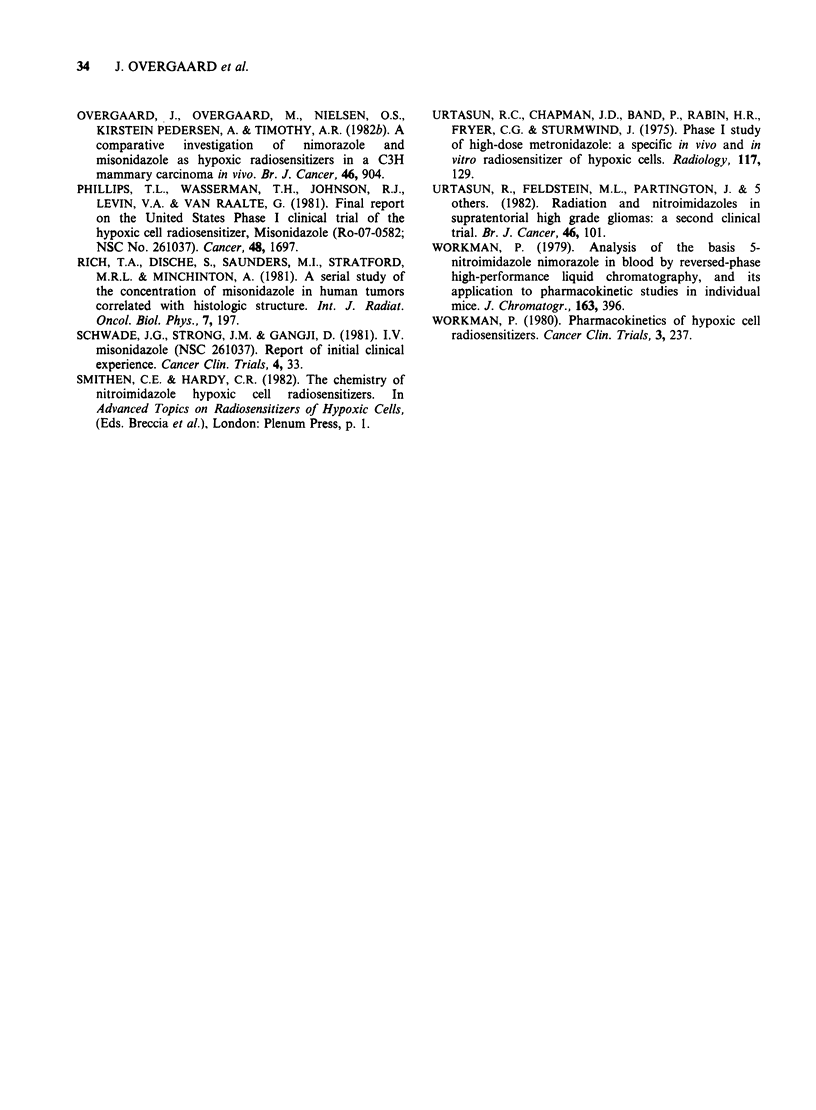# Studies of the pharmacokinetic properties of nimorazole.

**DOI:** 10.1038/bjc.1983.153

**Published:** 1983-07

**Authors:** J. Overgaard, M. Overgaard, A. R. Timothy

## Abstract

The pharmacokinetics of the hypoxic radio-sensitizer nimorazole were studied in 19 individuals after single oral doses of between 0.5-3.5 g. HPLC measurements showed, after a rapid absorption, a linear relationship between peak plasma concentration and given dose. Mean elimination half life was 3.1 h. A tendency to a dose-dependent variation in the apparent volume of distribution, total body clearance and elimination half life suggest non-linear pharmacokinetics of nimorazole. Tumour concentrations measured in 5 patients gave tumour/plasma ratios between 0.8-1.3. No toxicity was observed. The results indicate that nimorazole may have potential as a clinically useful hypoxic radiosensitizer.


*Br. J. Cancer* (1983), **48**, 27–34

### Studies of the pharmacokinetic properties of nimorazole

J. Overgaard[1], M. Overgaard[1] & A.R. Timothy[2]

[1]*Institute of Cancer Research and Department of Oncology and Radiotherapy, Radiumstationen, DK-8000 Aarhus C, Denmark and* [2]*Department of Radiotherapy, St. Thomas' Hospital, London SE1.*

**Summary** The pharmacokinetics of the hypoxic radio-sensitizer nimorazole were studied in 19 individuals after single oral doses of between 0.5–3.5 g. HPLC measurements showed, after a rapid absorption, a linear relationship between peak plasma concentration and given dose. Mean elimination half life was 3.1 h. A tendency to a dose-dependent variation in the apparent volume of distribution, total body clearance and elimination half life suggest non-linear pharmacokinetics of nimorazole. Tumour concentrations measured in 5 patients gave tumour/plasma ratios between 0.8–1.3. No toxicity was observed. The results indicate that nimorazole may have potential as a clinically useful hypoxic radiosensitizer.

Recent studies in experimental tumours *in vivo* suggest that nimorazole (1-(N-β-ethyl-morpholine)-5-nitro-imidazole) may possess hypoxic cell radiosensitizing properties similar to that of misonidazole (MISO) when given in moderate doses (Overgaard *et al.*, 1982*b*). At tumour concentrations of $\sim 50\,\mu g\,g^{-1}$ both drugs were found to yield enhancement ratios of ~1.4 in a C3H mammary carcinoma (Overgaard *et al.*, 1982*b*). Clinical studies of plasma and tumour concentrations indicate that an equivalent dose level in humans would require $\sim 1.5$–$2.0\,g\,m^{-2}$ MISO on the likely assumption that a tumour-plasma ratio of about unity can be achieved (Ash & Schmidt, 1980; Dische, 1982; Rich *et al.*, 1981). Such a dose cannot be given daily in conjunction with conventional radiation fractionation schedules due to excessive dose-limiting toxicity, primarily expressed as peripheral neuropathy (Dische *et al.*, 1978; Kogelnik 1980; Phillips *et al.*, 1981). Thus, accumulated doses >11–$12\,g\,m^{-2}$ MISO over a 4-week period carry a high risk of unacceptable side effects (Dische *et al.*, 1978, 1979; Kogelnik, 1980; Overgaard *et al.*, 1982*a*; Phillips *et al.*, 1981; Urtasun *et al.*, 1982).

Clinical experience with nimorazole as an antimicrobial agent indicates that the drug is considerably less toxic than MISO. Total doses of 25 g in 10 days have easily been administrated to both adults and children, the only side effect being mild gastrointestinal disturbances with nausea and occasional vomiting. This toxicity was observed in 5–25% of patients, was found to be dose-independent and often transitory and could otherwise be controlled with antiemetics (Arrubarrena *et al.*, 1971; Cué & Traslosheros, 1969; Daza, 1973; Garcia *et al.*, 1975; Oliva *et al.*, 1972).

Experience from large animal toxicity studies (Farmitalia, Carlo Erba, unpublished observations) suggests that nimorazole may be even better tolerated than metronidazole (Flagyl) which itself can be administrated in daily doses of $6\,g\,m^{-2}$ up to a total of ~100 g, although such high doses may be associated with significant gastrointestinal toxicity and some central and peripheral neuropathy (Kapp *et al.*, 1982; Karim, 1978; Urtasun *et al.*, 1975, 1982). If the lack of significant toxicity is confirmed, it may be possible to give nimorazole in conjunction with conventional daily radiation fractionation schedules at a dose level which may enhance the radiation response of hypoxic cells by a factor of ~1.4. Therefore, nimorazole represents a potentially attractive compound for hypoxic radiosensitization in clinical radiotherapy.

Although there is information about the tolerance and metabolism of single dose nimorazole (Giralsi *et al.*, 1971), details of the pharmacokinetic properties with regard to absorption, elimination and plasma and tumour concentrations are very limited. The present study was therefore performed with the aim of obtaining further information about the pharmacokinetics of nimorazole given in single doses between 0.5–3.5 g.

### Materials and methods

Pharmacokinetic measurements were performed in informed volunteers of whom 11 were patients receiving radiotherapy for malignant tumours, and 8 were healthy male scientists (Table I). All subjects had normal hepatic and renal function.

Nimorazole was supplied by Farmitalia, Carlo Erba in tablets of 500 mg which were given in single oral doses, as indicated in Table II. The drug was normally given with, or shortly after, a light breakfast. Serial venous blood samples were usually collected at intervals of 15–30 min over the first 2–3 h and then at 4, 6–8 and 24 h after administration.

Correspondence: J. Overgaard

Received 18 March 1983; accepted 11 April 1983

© The Macmillan Press Ltd., 1983

**Table I** Details of subjects in the study

| *Subject* | *Sex* | *Age (yr)* | *Weight (kg)* | *Height (cm)* | *Surface area ($m^2$)* | *Diagnosis* |
|---|---|---|---|---|---|---|
| 1 | M | 35 | 70 | 181 | 1.9 | Healthy |
| 2 | M | 29 | 67 | 177 | 1.9 | Healthy |
| 3 | F | 61 | 59 | 165 | 1.6 | Breast carcinoma |
| 4 | M | 36 | 90 | 196 | 2.3 | Healthy |
| 5 | M | 39 | 71 | 176 | 1.9 | Healthy |
| 6 | M | 32 | 79 | 185 | 2.1 | Healthy |
| 7 | M | 49 | 70 | 187 | 2.1 | Healthy |
| 8 | F | 65 | 65 | 154 | 1.6 | Rectal carcinoma |
| 9 | M | 63 | 49 | 168 | 1.5 | Epidermoid carcinoma of the pinna |
| 10 | M | 65 | 101 | 176 | 2.2 | Epidermoid carcinoma of the anus |
| 11 | M | 39 | 70 | 172 | 1.8 | Healthy |
| 12 | M | 76 | 55 | 170 | 1.6 | Prostate carcinoma |
| 13 | M | 50 | 55 | 173 | 1.6 | Epidermoid carcinoma of the larynx |
| 14 | M | 25 | 76 | 189 | 2.1 | Healthy |
| 15 | F | 72 | 75 | 163 | 1.8 | Lymphoma |
| 16 | M | 58 | 64 | 170 | 1.7 | Myeloma |
| 17 | M | 71 | 80 | 177 | 1.9 | Lymphoma |
| 18 | F | 56 | 64 | 170 | 1.7 | Malignant melanoma |
| 19 | M | 30 | 73 | 178 | 2.0 | Malignant melanoma |

No specific restrictions were given with regard to drinking or eating in the test period. In subjects receiving multiple doses, these were given with at least 4 weeks' interval.

*Measurement of drug*

Plasma and tumour concentrations of nimorazole were measured by high pressure liquid chromatography (HPLC). The venous blood was collected in heparinized tubes, centrifuged at 3,000 *g* for 10 min after which the plasma was removed. Methanol (250 μl) was added to 25 μl plasma together with naproxen as internal standard. After Vortex mixing the samples were allowed to rest for 10 min before centrifugation at 3,000 *g* for 5 min. The supernatant was collected in a Hamilton syringe and used for HPLC determination. A Waters high performance liquid chromatography system was used composed of 2 model 6000 A pumps, a model 660 solvent flow programmer, a model U6K injector, a model 440 fixed wavelength UV detector having an 8 μl flow through cell. The system was connected to an Omniscribe dual channel chart recorder. Reversed phase HPLC measurements were performed by injecting 25 μl of the sample onto a nucleosil 10 μ C18 column using 60% methanol in 20 mM phosphate buffer, pH 6.5 as mobile phase at a flow of 2 ml $min^{-1}$. The UV-absorption was measured at 313 nm (Figure 1).

Tumour concentrations were measured in tissues obtained by biopsy. After weighing a known amount of internal standard was added together with methanol to a 5-fold increase in volume. After mixing with a rotating knife, the sample was centrifuged at 3,000 *g* for 10 min. The supernatant was removed and the solvent evaporated at 37°C under a stream of dry nitrogen. Dried residue was redissolved in 500 μl methanol and after Vortex agitation, injected onto the HPLC column and estimated similar to the plasma samples. All plasma and tumour measurements were performed in duplicate, and the daily standard calibration curve was performed using different but known concentrations of drugs in plasma.

*Pharmacokinetic calculations*

The plasma pharmacokinetic parameters are described on the assumption of first order absorption and elimination kinetics. The elimination rate constant $k_e$ is determined during the elimination phase by least square regression calculation of the slope of a log-linear plot of nimorazole dose *versus* time. The elimination half life is then given by $\ln 2/k_e$. The area under the curve ($AUC_{0-\infty}$) which gives an expression of the concentration × time exposure to the drug was calculated by the trapezoidal rule from time 0 to time $t$ (usually 6 h). The remaining area was

**Figure 1** HPLC pattern of plasma containing 79 $\mu g\, ml^{-1}$ nimorazole extracted with methanol-containing naproxen as internal standard.

calculated by the formula $C_t/k_e$, where $C_t$ is the plasma concentration at time $t$. The apparent volume of distribution ($V_d$) was estimated as $D$/AUC where $D$ is the total dose in g. Plasma clearance was calculated as $D/k_e \cdot$AUC. Under the assumption that the kinetics follow a two-compartment one way model, a rough estimate of the absorption rate ($k_a$) is obtained by the formula $AUC = C_0/k_e - C_0/k_a$, where $C_0$ is the dose at time 0 obtained by extrapolation of the log-linear elimination curve. Similarly, the absorption half life was estimated as $\ln 2/k_a$.

### Results

Nimorazole was well tolerated, and with the exception of slight nausea in one subject (2.5 g dose), no acute or chronic side effects have been recorded.

Plasma pharmacokinetic parameters obtained after single doses from 0.5–3.5 g are shown in Table II and typical plasma profiles are illustrated in Figure 2. Nimorazole is easily absorbed after oral administration with a mean absorption half life of 24 min $\pm 2$ (s.e.). The absorption appears to be dose-independent. Peak plasma concentrations are reached between 35 and 125 min after intake (median 90 min). The peak plasma concentration was linear–linear related to the given dose in g, and this correlation was further improved if the dose was expressed in $g\, m^{-2}$ or better as $mg\, kg^{-1}$ (Figure 3). Thus, the peak plasma concentration was found to be $20.0 \pm 2.0$ (s.e.), $38.0 \pm 2.8$ and 1.30 $\pm 0.10\, \mu g\, ml^{-1}$, respectively when calculated per g, $g\, m^{-2}$ and $mg\, kg^{-1}$, respectively. Plasma elimination occurred with a half life ranging from 2.0–4.6 h with a mean of $3.1 \pm 0.1$. The elimination half life was found to be one of the most variable parameters but was not significantly altered as a function of dose (Figure 5).

The exposure to the drug calculated as the area under the curve (AUC) was also found to be dose related, although to a smaller extent than the peak plasma concentration due to the variations in drug elimination. However, there was a well-established linear–linear relationship between AUC and given dose, especially when expressed as $mg\, kg^{-1}$ (Figure 4).

The distribution of the drug estimated as the apparent volume of distribution ($V_d$) had a mean value of $0.79 \pm 0.04\, l\, kg^{-1}$. Although not statistically significant there appeared to be a trend towards a lower volume of distribution with increasing dose (Figure 5). A similar tendency was seen for the total body clearance (mean $218 \pm 19\, l\, min^{-1}$) which also appeared, although not statistically significant, to become smaller with increasing given dose. Thus, from the present data there are suggestions that certain parameters follow non-linear pharmacokinetics. This suggestion gets further support when analysing two subjects (1 and 4) in which multiple dose levels have been measured. Both showed an increasing elimination half life and a decrease in total body clearance and $V_d$ with increasing given doses (Table II). It is therefore likely that the pharmacokinetics may be non-linear on an individual basis, but that this non-linearity is so small that it requires a larger investigation before it becomes generally apparent.

From the point of hypoxic radiosensitizing effect, tumour rather than the plasma concentration is the

**Table II** Summary of plasma pharmacokinetic parameters

| Subject | Dose g | Dose $g m^{-2}$ | Dose $mg kg^{-1}$ | Peak conc. ($\mu g ml^{-1}$) | Reaction (min) | Absorption half life (min) | Elimination half life (h) | Plasma clearance ($ml min^{-1}$) | $V_d$ ($l kg^{-1}$) | $AUC_{0-\infty}$ ($\mu g ml^{-1} \cdot hr$) |
|---|---|---|---|---|---|---|---|---|---|---|
| 1 | 0.5 | 0.3 | 7.1 | 5.9 | 60 | 22 | 2.6 | 320 | 1.03 | 26 |
| 2 | 0.5 | 0.3 | 7.5 | 7.0 | 60 | 22 | 2.5 | 278 | 0.90 | 30 |
| 3 | 1.0 | 0.6 | 16.9 | 14.7 | 120 | 49 | 3.7 | 183 | 0.99 | 91 |
| 4 | 1.0 | 0.4 | 11.1 | 10.0 | 65 | 20 | 2.4 | 417 | 0.96 | 40 |
| 5 | 1.5 | 0.8 | 21.1 | 23.5 | 35 | 11 | 2.9 | 240 | 0.85 | 104 |
| 6 | 1.5 | 0.7 | 19.0 | 29.2 | 100 | 23 | 2.7 | 187 | 0.55 | 134 |
| 1 | 1.5 | 0.8 | 21.4 | 24.6 | 60 | 44 | 2.8 | 248 | 0.86 | 101 |
| 7 | 1.5 | 0.7 | 21.4 | 19.0 | 100 | 46 | 2.0 | 397 | 0.98 | 63 |
| 8 | 1.5 | 0.9 | 23.1 | 25.7 | 90 | 31 | 3.2 | 182 | 0.77 | 138 |
| 4 | 2.0 | 0.8 | 22.8 | 22.4 | 120 | 25 | 3.1 | 273 | 0.80 | 122 |
| 9 | 2.0 | 1.3 | 40.0 | 49.8 | 60 | 18 | 3.5 | 113 | 0.69 | 293 |
| 10 | 2.0 | 0.9 | 19.8 | 31.0 | 60 | 16 | 2.9 | 233 | 0.58 | 143 |
| 11 | 2.0 | 1.1 | 28.6 | 39.1 | 100 | 24 | 4.6 | 108 | 0.62 | 310 |
| 12 | 2.0 | 1.3 | 35.7 | 56.9 | 60 | 13 | 2.6 | 140 | 0.57 | 238 |
| 13 | 2.0 | 1.2 | 36.4 | 44.2 | 90 | 12 | 3.6 | 125 | 0.71 | 266 |
| 14 | 2.5 | 1.2 | 32.9 | 34.4 | 60 | 17 | 3.5 | 210 | 0.84 | 199 |
| 15 | 2.5 | 1.3 | 33.3 | 25.0 | 60 | 23 | 3.0 | 372 | 1.29 | 109 |
| 2 | 2.5 | 1.3 | 37.3 | 50.5 | 60 | 8 | 3.5 | 145 | 0.66 | 285 |
| 16 | 3.0 | 1.7 | 46.9 | 69.5 | 90 | 32 | 4.2 | 107 | 0.61 | 469 |
| 1 | 3.0 | 1.6 | 42.9 | 49.4 | 120 | 25 | 3.2 | 168 | 0.67 | 296 |
| 4 | 3.0 | 1.3 | 33.3 | 41.0 | 125 | 41 | 3.7 | 192 | 0.69 | 262 |
| 12 | 3.5 | 2.2 | 63.6 | 79.3 | 45 | 14 | 2.9 | 155 | 0.71 | 376 |

**Figure 2** Typical plasma concentration profiles of nimorazole given in different doses.

**Figure 3** The relationship between dose of nimorazole in g, $gm^{-2}$ or mg $kg^{-1}$ and peak plasma concentration.

**Figure 4** Relationship between dose of nimorazole in $mgkg^{-1}$ and $AUC_{0-\infty}$.

critical tissue. Tumour biopsies were obtained from 5 patients ~2h after intake of the drug (Table III). The tumour/plasma ratios ranged from 0.84–1.30 indicating that the same concentration levels can be achieved in tumour and plasma. In a part of the tumour sample from subject 8 a low tumour concentration was obtained, but this sample consisted of necrotic fluid and in more solid tumour tissue from the same metastasis a considerably higher concentration was found.

Early in the study nimorazole was tested for its potential clinical radiosensitizing ability. The data in Figure 6 show the effect of nimorazole on the initial radiation response in metastases from a malignant melanoma. In the same patient, different cutaneous metastases were treated with single doses of radiation, either alone or 4h after a dose of nimorazole (51 $mgkg^{-1}$). This 4h interval between drug intake and radiation was because the treatment was performed prior to these

**Figure 5** Scatter diagram showing the relationship between dose of nimorazole in $mgkg^{-1}$ and the apparent volume of distribution ($V_d$), total body clearance and elimination half life.

**Table III** Comparable plasma and tumour concentrations

| Subject | Dose ($mg\,kg^{-1}$) | Time after intake (min) | Concentration Plasma ($\mu g\,ml^{-1}$) | Concentration Tumour ($\mu g\,g^{-1}$) | Tumour/plasma ratio | Biopsy |
|---|---|---|---|---|---|---|
| 10 | 19.8 | 135 | 18.7 | 19.8 | 1.06 | Local anal recurrence |
| 8 | 23.1 | 120 | 20.7 | 17.5 | 0.85 | Subcutaneous gluteal metastasis |
| 8 | 23.1 | 120 | 20.7 | <2 | <0.10 | Same, necrotic fluid |
| 17 | 25.0 | 100 | 14.2 | 14.1 | 0.99 | Subcutaneous chestwall metastasis |
| 18 | 31.3 | 120 | 17.0 | 14.3 | 0.84 | Cutaneous melanoma on leg |
| 9 | 40.0 | 120 | 32.2 | 41.9 | 1.30 | Neck node |

**Figure 6** Initial regression of cutaneous metastases from malignant melanoma treated with different single doses of radiation alone or radiation 4 h after a dose of 51 mg kg$^{-1}$ nimorazole. (Subject 19).

pharmacokinetic studies and used a schedule similar to that applied to MISO. Presuming that the dose response pattern for initial tumour regressing reflects the radiation response, nimorazole seems to result in an enhanced radiation response. Naturally such an assumption is subject to severe limitations, but is supported by the apparent dose-response relationship. No obvious differences in acute skin reaction at identical radiation dose levels was noted; however, the patient died 2 months after treatment due to disseminated disease.

### Discussion

The plasma pharmacokinetic analysis of nimorazole indicates that the drug is readily absorbed and that concentrations $>50\,\mu g\,ml^{-1}$ can be achieved in the plasma after doses of $1.5\,g\,m^{-2}$. With all the reservations inherent in attempts to extrapolate from experimental data to the clinical situation, it is reasonable to assume that such a concentration, if attainable in the tumour, may result in substantial hypoxic cell radiosensitization (Overgaard *et al.*, 1982*b*).

The elimination of nimorazole occurs with a half life of ~3 h compared to 10–12 h for MISO. In spite of that, the peak plasma concentrations observed in the present study are very similar to those obtained with comparable doses of oral MISO and metronidazole (Deutsch *et al.*, 1975; Dische *et al.*, 1979; Kogelnik, 1980; Phillips *et al.*, 1981; Schwade *et al.*, 1981; Workman, 1980). However, exposure to the drug expressed by the AUC was, at equivalent doses, only about one third of the AUC values for MISO (Overgaard *et al.*, unpublished observations), and this may account for the low toxicity. Thus, no neurological disturbances have been observed, the only side effect being gastrointestinal, and at a level which can be controlled by antiemetic therapy.

In addition, the tumour plasma ratios were similar to those observed with MISO (Rich *et al.*, 1981), and it is likely that the concentration in viable tumour tissue will be of the same magnitude as the plasma level. The low level found in necrotic tissue in one tumour is also in agreement with more detailed data from tumours exposed to MISO (Rich *et al.*, 1981). The apparently similar tumour/plasma ratios of MISO and nimorazole despite differences in elimination half life were an expected observation (Workman, 1980). It is therefore likely that tumour concentrations corresponding to those which result in an enhancement ratio of 1.4 in an experimental tumour treated with single doses of nimorazole can be achieved in the clinical situation. Furthermore, the experimental results indicate that the dose-response relationship for the radiosensitizing effect of nimorazole is minimal once a threshold has been reached (Overgaard *et al.*, 1982*b*). It is anticipated that this threshold level can be achieved clinically, since nimorazole appears to be no more toxic than metronidazole (Flagyl). Thus, it is expected that nimorazole could be given in daily doses of $\sim 1.5\,g\,m^{-2}$ over 5–6 weeks which will allow the drug to be used in conventional radiation fractionation schedules. An on-going phase 1 clinical study has shown that 2 g daily for 4 weeks can be tolerated without side effects other than moderate and acceptable nausea (Timothy *et al.*, unpublished observations).

The tendency to non-linear pharmacokinetics observed in the present study is not unexpected since similar patterns have been observed for both MISO and nimorazole in mice (Workman, 1979, 1980). However, the extent of the observed non-linearity is small and within the variations otherwise observed among the investigated subjects. It may therefore have no practical implication for the use of nimorazole as a hypoxic radiosensitizer.

Nimorazole is almost completely metabolized and two metabolites have been characterized (Giraldi, 1971; Workman, 1980). In the present study these have not been analysed, but preliminary studies suggest a lower hypoxic radiosensitizing ability than nimorazole and these may therefore not contribute significantly to the overall hypoxic radiosensitization (Smithen & Hardy, 1982).

The present pharmacokinetic data together with the experimental tumour studies (Overgaard *et al.*, 1982*b*) give promise of a hypoxic radiosensitizer that may be used clinically in doses which, when given in normal radiation fraction schemes, may result in higher enhancement ratios than any currently available sensitizer in clinical use, and with a low toxicity. Therefore, nimorazole deserves a high priority among those hypoxic cell radiosensitizers which are currently being considered as candidates for clinical evaluation.

Supported by the Danish Cancer Society, grant no. 24/79, and "Grosserer Sigurd Abrahamson og Hustru Addie Abrahamsons mindelegat".

### References

ARRUBARRENA, V.M., GUTIÉRREZ, C.A. & LÓPEZ, G.L. (1971). Tratamiento del absceso hepático amibiano con nitrimidazina. *Rev. Gastroent. Méx.*, **36**, 140.

ASH, D.V. & SMITH, M.R. (1980). Causes of apparent low levels of misonidazole in human tumours. *Clin. Radiol.*, **31**, 233.

CUÉ, A.B. & TRASLOSHEROS, J.C. (1969). The use of new amebacides in children (a preliminary report). *ISSSTE Revista Médica*, **8**, 1.

DAZA, M.C.G. (1973). Valoracion de la nitrimidazina en el tratamiento de la amibiasis. *Pensa Med. Mex.*, **38**, 145.

DEUTSCH, G., FOSTER, J.L., MCFADZEAN, J.A. & PARNELL, M. (1975). Human studies with "high dose" metronidazole: a non-toxic radiosensitizer of hypoxic cells. *Br. J. Cancer*, **31**, 75.

DISCHE, S. (1982). Misonidazole and desmethylmisonidazole in clinical radiotherapy. In *Advanced Topics on Radiosensitizers of Hypoxic Cells*, (Eds. Breccia, *et al.*). London: Plenum Press, p. 229.

DISCHE, S., SAUNDERS, M.I., ANDERSON, P. & 6 others. (1978). The neurotoxicity of misonidazole: pooling of data from five centres. *Br. J. Radiol.*, **51**, 1023.

DISCHE, S., SAUNDERS, M.I., FLOCKHART, I.R., LEE, M.E. & ANDERSON, P. (1979). Misonidazole—a drug for trial in radiotherapy and oncology. *Int. J. Radiat. Oncol. Biol. Phys.*, **5**, 851.

GARCIA, O.O., NAVA, J.P. & DIAZ, R.G. (1975). Valoracion clinica del nimorazol en absceso hepatico amibiano. *Prensa Med. Mex.*, **40**, 378.

GIRALDI, P.N., TOSOLINI, G.F., DRADI, E. & 5 others. (1971). Studies on antiprotozoans—III. Isolation, identification and quantitative determination in humans of the metabolites of a new trichomonacidal agent. *Biochem. Pharmacol.*, **20**, 339.

KAPP, D.S., WAGNER, F.C. & LAWRENCE, R. (1982). Glioblastoma multiforme: treatment by large dose fraction irradiation and metronidazole. *Int. J. Radiat. Oncol. Biol. Phys.*, **8**, 351.

KARIM, A.B.M.F. (1978). Prolonged metronidazole administration with protracted radiotherapy: a pilot study on response of advanced tumours. *Br. J. Cancer*, **37** (Suppl. III), 299.

KOGELNIK, H.D. (1980). Clinical experience with misonidazole. *Cancer Clin. Trials*, **3**, 179.

OLIVA, A.L., MANCILLAS, A.P. & ZAMORA, R.J. (1972). Tratamiento del absceso hepático amibiano y la amibiasis intestinal con Nitrimidazina y etofamida. *Rev. Medicina*, **52**, 563.

OVERGAARD, J., ANDERSEN, A.P., JENSEN, R.H. & 5 others. (1982*a*). Misonidazole combined with split-course radio-therapy in the treatment of invasive carcinoma of the larynx and the pharynx. *Acta oto-laryng.* (*Stockh.*), *Suppl.* **386**, 215.

OVERGAARD, J., OVERGAARD, M., NIELSEN, O.S., KIRSTEIN PEDERSEN, A. & TIMOTHY, A.R. (1982*b*). A comparative investigation of nimorazole and misonidazole as hypoxic radiosensitizers in a C3H mammary carcinoma *in vivo*. *Br. J. Cancer*, **46**, 904.

PHILLIPS, T.L., WASSERMAN, T.H., JOHNSON, R.J., LEVIN, V.A. & VAN RAALTE, G. (1981). Final report on the United States Phase I clinical trial of the hypoxic cell radiosensitizer, Misonidazole (Ro-07-0582; NSC No. 261037). *Cancer*, **48**, 1697.

RICH, T.A., DISCHE, S., SAUNDERS, M.I., STRATFORD, M.R.L. & MINCHINTON, A. (1981). A serial study of the concentration of misonidazole in human tumors correlated with histologic structure. *Int. J. Radiat. Oncol. Biol. Phys.*, **7**, 197.

SCHWADE, J.G., STRONG, J.M. & GANGJI, D. (1981). I.V. misonidazole (NSC 261037). Report of initial clinical experience. *Cancer Clin. Trials*, **4**, 33.

SMITHEN, C.E. & HARDY, C.R. (1982). The chemistry of nitroimidazole hypoxic cell radiosensitizers. In *Advanced Topics on Radiosensitizers of Hypoxic Cells*, (Eds. Breccia *et al.*), London: Plenum Press, p. 1.

URTASUN, R.C., CHAPMAN, J.D., BAND, P., RABIN, H.R., FRYER, C.G. & STURMWIND, J. (1975). Phase I study of high-dose metronidazole: a specific *in vivo* and *in vitro* radiosensitizer of hypoxic cells. *Radiology*, **117**, 129.

URTASUN, R., FELDSTEIN, M.L., PARTINGTON, J. & 5 others. (1982). Radiation and nitroimidazoles in supratentorial high grade gliomas: a second clinical trial. *Br. J. Cancer*, **46**, 101.

WORKMAN, P. (1979). Analysis of the basis 5-nitroimidazole nimorazole in blood by reversed-phase high-performance liquid chromatography, and its application to pharmacokinetic studies in individual mice. *J. Chromatogr.*, **163**, 396.

WORKMAN, P. (1980). Pharmacokinetics of hypoxic cell radiosensitizers. *Cancer Clin. Trials*, **3**, 237.